# Economic feasibility and risk analysis of industrial hemp production: a comparative assessment of floral, fiber, and grain enterprises in North Carolina, USA

**DOI:** 10.1186/s42238-025-00364-x

**Published:** 2025-12-08

**Authors:** Obed Quaicoe, Fafanyo Asiseh, Atta Aloka, Omoanghe Isikhuemhen, Felicia Anike

**Affiliations:** 1 Department of Agribusiness, Applied Economics and Agriscience Education, College of Agriculture and Environmental Sciences, North Carolina Agricultural and Technical State University, 1601 E. Market St, Greensboro, NC 27411 USA; 2 Department of Economics, College of Business and Economics, North Carolina Agricultural and Technical State University, 1601 E. Market St, Greensboro, NC 27411 USA; 3 Department of Natural Resources, College of Agriculture and Environmental Sciences, North Carolina Agricultural and Technical State University, 1601 E. Market St, Greensboro, NC 27411 USA

**Keywords:** Hemp production, Enterprise budget, Benefit–cost analysis, Net present value, Internal rate of return, Monte Carlo simulation

## Abstract

This study evaluates the economic viability of industrial hemp production in North Carolina, focusing on three production systems: floral, grain, and fiber. Enterprise budgets were developed to assess costs and returns per hectare. Risk-based investment metrics, including net present value (NPV), internal rate of return (IRR), and benefit–cost ratio (BCR), were projected over a five-year horizon. Results indicate that floral hemp has the highest estimated production costs but also offers the greatest potential, while fiber and grain hemp present more moderate but stable outcomes. Estimated IRRs for all three systems exceeded the 5.5% discount rate, and BCRs were above one, suggesting that revenues can cover costs under baseline conditions. Sensitivity analyses highlight the influence of yield and market price variability, particularly for floral hemp, which shows higher upside potential, but greater risk compared to grain and fiber. Monte Carlo simulations reinforce these findings, demonstrating that profitability is possible across all hemp types but subject to significant uncertainty, especially in floral markets. The analysis also reveals that limited processing capacity and the absence of equilibrium pricing in North Carolina contribute to market inefficiencies, constraining producer profitability. Overall, these results provide benchmark financial estimates for growers and policymakers while the region’s hemp markets, and processing infrastructure continue to mature.

## Introduction

Industrial hemp (*Cannabis sativa* L.) has re-emerged in the United States (U.S.) as a multifunctional crop valued for its applications in textiles, construction materials, and food products. This resurgence was primarily stimulated by regulatory reforms and growing consumer demand (Mark et al. 2020) and was further reinforced by perceptions that hemp offered higher economic returns compared to other crops (Mark and Will 2019; Quaicoe et al. 2023). The 2014 and 2018 U.S. Farm Bills established a national pilot program and subsequently legalized commercial production, triggering rapid expansion but also exposing the industry to cycles of oversupply and price collapse (Mark et al. 2020). These boom-and-bust dynamics highlight the importance of examining not only the agronomic potential of hemp but also the economic forces that shape producer profitability, processor capacity, and overall market stability.

**Fig. 1:**
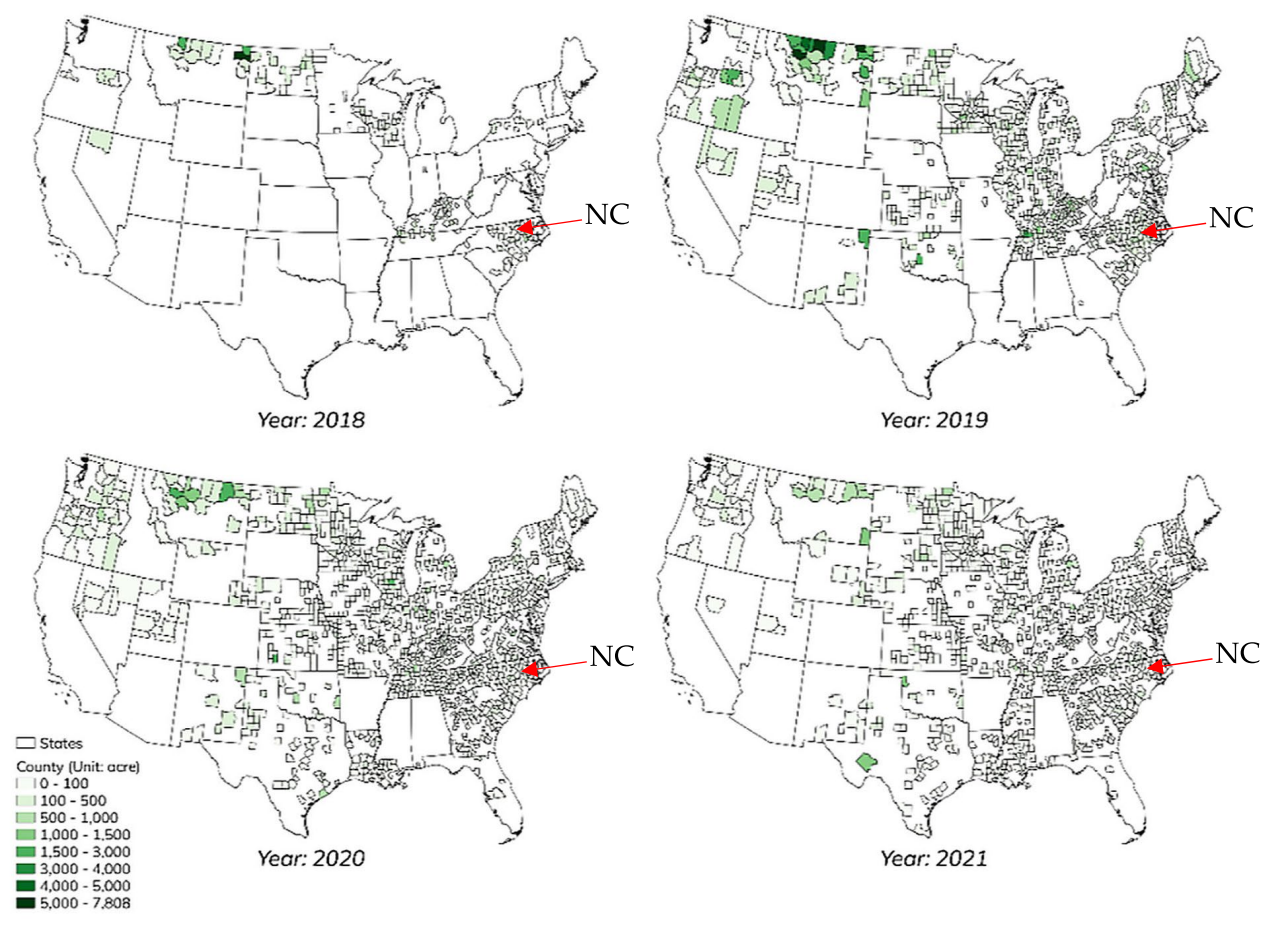
Planted area of Hemp Production in the U.S (State-level). Source: Tyler Mark (2022). Hemp Market Structure, Pricing, and Sustainability. USDA GRANT13669690/2022–06904

Within this context, North Carolina (N.C.) represents a compelling case. As one of the top ten hemp-producing regions in the U.S., North Carolina. has experienced both promising opportunities and persistent market inefficiencies (Lambert and Hagerman 2022). The market is promising in terms of long-term potential but currently underdeveloped in infrastructure and marketing channels (Lambert and Hagerman 2022). North Carolina began issuing hemp licenses in 2017, and cultivation expanded swiftly thereafter; between 2017 and 2021, the number of licensed hemp growers rose by more than 1,100% (Quaicoe et al. 2023), while cultivated areas peaked in 2019 and 2020 before contracting in subsequent years (Fig. 1). According to industry data and the U.S. hemp survey, hemp cultivation expanded from less than 40,500 hectares in 2018 to over 202,300 hectares in 2019, before contracting to less than 20,200 hectares by 2021 (National 2022).

More recently, the United States Department of Agriculture (USDA) reported that North Carolina’s total planted hemp area in 2023 was slightly lower than the previous season, yet open-field production increased sharply—133% for fiber, 18% for flowers, and 28% for grain (Lambert and Hagerman 2022). These trends illustrate the volatility of the sector, where farmers often entered production because of the novelty of the crop and the promise of high returns, but many lacked sufficient awareness of the associated financial risks (Quaicoe et al. 2023). The consequences were evident in 2018–2019, when fiber and floral output exceeded market demand, resulting in widespread losses (Mark et al. 2020). Since legalization in 2014, the hemp industry has been shaped by cyclical fluctuations influenced by regulatory complexity, uncertain consumer demand, and limited processing capacity (Dhoubhadel 2021). The 2019 price bubble for floral hemp along with continuing supply chain inefficiencies (Lambert and Hagerman 2022), underscores the need for deeper insight into the economic and financial dynamics of hemp cultivation. While end-use markets continue to expand and federal reforms have improved price prospects, particularly for floral hemp (Lambert and Hagerman 2022), underdeveloped infrastructure and unsold inventories remain persistent challenges (Hill et al. 2023; Shepherd et al. 2024).

The structure of the global industrial hemp market has undergone significant transformations over time. Despite ongoing challenges, global demand projections underscore hemp’s long-term potential (Kaur and Kander 2023; Quaicoe et al. 2020; Yano and Fu 2023). The industrial hemp market was valued at USD 5.99 billion in 2024 and is forecast to reach USD 34.16 billion by 2034, with compound annual growth rate (CAGR) of 19% (). Canada leads in hemp-based foods, China supplies nearly half of global hemp fiber, and France dominates EU production (Victoria State Government 2020; McGrath 2019; Hemp 2025). Growth is further supported by favorable policies, rising demand in textiles, and rapid expansion in the Asia–Pacific region, where access to raw materials and global market linkages are strong (Expert 2025). Looking forward, applications in biofuels, bioplastics, and medical research are projected to sustain market expansion (Markets 2025). Considering these challenges and opportunities, this study evaluates the economic feasibility and risk profile of hemp cultivation in North Carolina using key financial metrics and Monte Carlo probabilistic simulations. By examining profitability, price volatility, and investment risk, the analysis provides critical insights into the sustainability of hemp farming. These findings aim to guide producers, investors, and policymakers in making informed decisions that support the long-term growth of the hemp industry in North Carolina.

Although opportunities for hemp research have expanded rapidly in recent years (Mark et al. 2020; Adesina et al. 2020; Cherney and Small 2016; Aydoğan et al. 2020; Wylie et al. 2021), knowledge of the economic challenges of cultivating industrial hemp and the quantitative assessments of hemp’s economic potential in the U.S. remains limited (Mark et al. 2020; Antle and Cho 2025). The literature has not yet offered a regional evaluation of hemp’s economic potential for integration into existing production systems in North Carolina at a commercial scale, nor has it applied methods that jointly assess economic feasibility and probabilistic risk in this potential hemp-producing region. While a few studies have evaluated the economic viability of hemp production (Michels et al. 2025; Budhathoki et al. 2024; Barnes et al. 2023; Khanal and Shah 2024), a recent systematic review of 98 published studies confirms that profitability assessments of hemp products remain a significant gap in literature (Budhathoki et al. 2024). This study contributes to the growing body of literature on the profitability of industrial hemp in several important ways. While previous research (Mark et al. 2020; Michels et al. 2025; Budhathoki et al. 2024; Barnes et al. 2023; Khanal and Shah 2024) and regional enterprise budgets have examined hemp profitability in selected regions of the U.S., there remains limited scholarship that integrates both economic feasibility and probabilistic risk analysis at the regional level. This gap is particularly evident in North Carolina which is one of the major but underexplored hemp-producing regions in the U.S. Existing studies have largely emphasized estimates of costs and returns, without explicitly quantifying the financial risks and volatility associated with market fluctuations, regulatory uncertainty, and supply chain constraints. To address this, the present study provides the first comprehensive economic and risk assessment of hemp cultivation in North Carolina, encompassing floral, fiber, and grain production systems. The study applies Monte Carlo probabilistic simulations to capture uncertainty in prices, yields, and input costs across three production systems (floral, grain, and fiber). This approach provides a more rigorous and robust characterization of financial outcomes and profitability thresholds than traditional static enterprise analyses. By combining economic evaluation with risk modeling in a regionally focused context, the study advances understanding of the sustainability and financial viability of hemp production in North Carolina extending beyond prior research that primarily documents profitability without adequately accounting for risk.

### Brief outlook of North Carolina agriculture and industrial hemp production

North Carolina’s agricultural sector is both diverse and economically significant to the U.S. As of 2023, the region ranked among the top ten national producers and exporters of several agricultural products including tobacco, hemp, sweet potatoes, and nursery crops (Government 2024; United States Department of Agriculture 2024). Field crops cover more than 1.5 million hectares, while livestock and poultry production contributes approximately 60% of total farm cash receipts in the region (USDA, NASS 2024). Nearly 47% of North Carolina farms cultivate labor-intensive row crops that demand considerable manual labor and mechanization (United 2022). The labor demand and other resource requirements of N.C agriculture make the integration of industrial hemp a practical addition to the region’s existing farming systems. For example, many tobacco growers in North Carolina view hemp, particularly floral hemp, as a viable diversification option for their operations, given its compatibility with existing tobacco production systems (Quaicoe et al. 2023). This allows tobacco farmers to leverage current resources, including transplanters, labor, and curing barns, thereby reducing the need for significant new capital investments (Quaicoe et al. 2023).

**Fig. 2:**
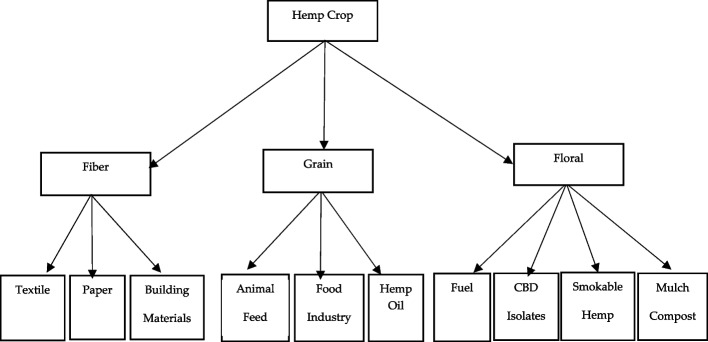
Principal products and uses derived from industrial-hemp plant parts. Source: author-generated schematic based on Kaur et al. (2025)

Hemp cultivation offers numerous benefits (Kaur and Kander 2023; Rupasinghe et al. 2020; Bridgeman and Abazia 2017; Sgrò et al. 2021). Hemp plants, grains, flowers, stalks, stems, and roots serve as raw materials for generating a wide range of valuable products (Fig. 2).

Hemp fibers, derived from stalk bast, can be used to manufacture a wide range of products, including textiles, clothing, rope, and even building materials (Gedik and Avinc 2020). Hemp grains are nutritional powerhouses that are rich in protein, healthy fats, and essential minerals and have applications in food and oil production (Crini et al. 2020a; Ahmed et al. 2022; Karche 2019; Promhuad et al. 2022). The flowers and leaves hold promise for the extraction of cannabidiol (CBD), a non-psychoactive compound for potential therapeutic, health, and wellness, extracts, oils, topicals, aromatic products, etc. (Cherney and Small 2016; Mileti et al. 2023; Szalata et al. 2022). Smokable hemp, defined as trimmed floral biomass containing less than 0.3 percent delta-9-tetrahydrocannabinol (THC), constitutes a rapidly growing inhalation market segment. The hemp roots are also being explored for pain management and other therapeutic applications (Schumacher et al. 2020; Herppich et al. 2020; Mańkowski et al. 2020). Environmentally, the hemp crop is a promoter of soil health. The deep root systems of hemp promote soil aeration, prevent erosion, and suppress weeds, fostering a thriving ecosystem (Schumacher et al. 2020; Mańkowski et al. 2020; Fernando et al. 2015). Post-extraction inflorescences can also be composted or applied as on-farm mulch, returning organic matter to the soil. Furthermore, hemp is a water-wise crop that requires significantly less water than traditional fibers such as cotton (Yano and Fu 2023). Hemp cultivation also acts as a carbon sink, absorbing atmospheric carbon dioxide at a rapid rate and potentially mitigating the effects of climate change (Pretot et al. 2014; Crini et al. 2020b).

Despite its economic and environmental benefits, hemp cultivation faces significant challenges (Basak et al. 2025). Regulatory challenges often involve complex licensing procedures and testing requirements, creating a hurdle for potential farmers (Dhoubhadel 2021). Finding markets to sell hemp products remains a major problem for producers (Biely and Passel 2022). Producers are still struggling to secure stable markets for their hemp products. The lack of infrastructure for processing hemp products and failure to uphold contractual obligations between hemp farmers and processors have contributed to inefficiencies and instability in the industrial hemp market (Quaicoe et al. 2023). The inefficiency and instability in the industrial hemp market inhibits the proper functioning of the market and hinders the sustainability of the hemp industry (Biely and Passel 2022).

As in many other parts of the world (Hemp - European Commission 2025; Mooney and Hill 2023; Jenner 2025), access to credit and startup capital remains a significant challenge for many North Carolina farmers interested in cultivating industrial hemp. Undercapitalization is a major hurdle, particularly regarding cultivating floral hemp, which is a costly crop to grow (Dhoubhadel 2021). Many farmers have relied on funding sources such as personal savings, retirement, cooperatives, and loans from friends and family for adequate funds to cover expenses and operate a successful hemp business (Quaicoe et al. 2023). Insufficient funding hinders the level of support necessary for sustainable agriculture (DeLonge et al. 2020, 2016; Barbosa 2024; Rodriguez et al. 2009). Additionally, farmers lack sufficient agronomic knowledge about various aspects of hemp production, including appropriate seed density, processing techniques, equipment selection, pests, and diseases (Caldwell et al. 2025). A lack of understanding can decrease yield, increase cost, and lower profit (Devkota et al. 2019). Although hemp crops have the potential to provide opportunities to farmers and other stakeholders in North Carolina, the economic and financial sustainability of the hemp industry is a major concern (Mark et al. 2020; Antle and Cho 2025; Budhathoki et al. 2024). Many farmers were motivated by the perceived market opportunities and entered hemp production too quickly without fully understanding the crop’s financial economics (Quaicoe et al. 2023).

## Materials and methods

For this study, a combination of representative data sources and economic modeling were used to evaluate the economic feasibility of producing hemp for flowers, fibers, and grains. Data from 2022 to 2024 were sourced from the USDA National Agricultural Statistical Service (National 2022; United States Department of Agriculture 2024; USDA, NASS 2024; United 2022). Compared to other agricultural commodities, national and regional data on hemp production are relatively limited (Quaicoe et al. 2023; Antle and Cho 2025). Furthermore, state-level data on hemp cultivated for grain or seed are currently unavailable, as this production segment remains in its early stages of development in many U.S. states, including North Carolina (United States Department of Agriculture 2024). As a result, quantitative assessments of hemp’s economic potential are constrained by the lack of historical yield and cost information at both the farm and market levels, making rigorous economic evaluation challenging (Antle and Cho 2025). The first comprehensive national and state-level data on hemp yields, hectares, and prices were released in 2022 (Antle and Cho 2025; National 2022). Most of the more detailed economic information currently available comes from enterprise budgets, which are primarily designed to help growers evaluate potential profitability (Antle and Cho 2025; North 2025; Seavert and Crabtree 2021). Additional studies provide data estimates of production costs and yields either through engineering–economic modeling (Barnes et al. 2023; Schumacher et al. 2020; Das et al. 2017) or from surveys of a relatively small number of producers (Antle and Cho 2025; Dingha et al. 2019).

The economic model used in this study hypothesizes that hemp growers aim to maximize their expected net returns. This suggests that a hemp grower can quit if the hemp enterprise is not yielding optimal profits. The assumptions regarding hemp growers' expectations are based on average weather conditions. Production input and product prices are considered exogenous, and the expected net returns are calculated, assuming an increasing return to scale (IRS) within the production system since industrial hemp production is an emerging industry in North Carolina. In this study, the decision-making process of hemp growers is framed as a problem of profit maximization, with the assumption that there are no constraints on the profits achievable by hemp growers. Assuming no constraints on profit serves as a benchmark framework that abstracts from real-world limitations such as capital scarcity, labor availability, regulatory compliance, and production risk. This approach allows for the isolation and examination of fundamental economic trade-offs prior to the incorporation of more complex, context-specific constraints.

To evaluate the financial and economic viability of hemp cultivation, net present value (NPV), the benefit‒cost ratio (BCR), and the internal rate of return (IRR) metrics (Szalata et al. 2022) were used. NPV was used to determine whether a hemp farmer should consider making a long-term investment in hemp cultivation. The NPV was estimated via Eq. (1). This estimation incorporated the initial investment and all future revenues and expenses over five years.

1 $$NPV=\sum\nolimits_{t=0}^{5}\frac{{(B}_{t}- {C}_{t})}{(1+r{)}^{t}}$$

where ${B}_{t}$ and ${C}_{t}$ represent the annual total benefits and total investment cost in year *t*, respectively; and $r$ is the assumed discount rate. A positive NPV suggests that, over the long term, a hemp farmer is likely to profit from investing in hemp cultivation after accounting for the time value of money, whereas a negative NPV indicates a reduction in the value of the investment. The IRR was utilized to assess the potential profitability of investing in hemp production. IRR represents the interest rate at which the discounted cash inflows from the production system exactly match the initial investment and ongoing costs. The discount rate at which the NPV equals zero was estimated using Eq. (2).

2 $$0=NPV=\sum\nolimits_{t=0}^{5}\frac{{(R}_{t}- {C}_{t})}{(1+i{)}^{t}}$$

where ${B}_{t}$ and ${C}_{t}$ represent the annual total benefits and total investment cost in year *t*, respectively; and *i* is the IRR (discount rate at which NPV = 0). The root-finding algorithm in R statical software determines the discount rate that equates Eq. (2) to zero, thereby enabling direct comparison with NPV estimates. An investment is deemed feasible if the estimated IRR exceeds the interest rate or borrowing cost of the investment. BCR was used to evaluate investment risk by comparing the total discounted expected benefits to the total discounted costs of hemp production, as shown in Eq. (3).

3 $$BCR=\frac{\sum_{t=0}^{5}\frac{{B}_{t}}{(1+r{)}^{t}}}{\sum_{t=0}^{5}\frac{ {C}_{t}}{(1+r{)}^{t}}}$$

where ${B}_{t}$ represents the discounted benefit in period *t*; ${C}_{t}$ represents the discounted cost in period *t*; *t* is time; and $r$ is the discount rate. In the BCR estimation, the total initial investment cost was incorporated into the period zero cost stream and included in the denominator’s sum of discounted costs in Eq. (3). This approach ensures consistency in cost accounting between BCR and NPV calculations.

Sensitivity analyses were performed to model the effects of market price fluctuations and yield variations on profits by applying ± 10% and ± 20% shocks to hemp market prices and yields, representing moderate and severe stress scenarios, respectively. This involved multiple iterations of changes in the market price and yield to calculate the adjusted revenues and profits. Additionally, a Monte Carlo probabilistic simulation with 10,000 iterations was used to generate probability distributions for NPV, enabling an assessment of the financial risks and potential impacts associated with market price volatility and production uncertainties. This simulation combined lognormal distributions for prices and yields with triangular distributions for production costs. To model realistic joint behavior of yield and price, a Gaussian copula with a correlation coefficient ($\rho$) of –0.3 was used. This parameter reflects a mild inverse relationship between yield and price shocks in crop markets, where high yields tend to exert downward pressure on prices, and low yields tend to drive prices up (Ramsey et al. 2019; Stuart et al. 2023).

From the simulation model, full distributions of net present value (NPV) were obtained and analyzed, using summary statistics and quantile-based measures, to evaluate downside risk exposure. To address the inherent uncertainty in key economic parameters such as crop prices and production costs for hemp, simulations were conducted not only for a representative base case but also across a broad range of alternative values. Economic outcomes were compared across three 5-year planning scenarios. The baseline scenario used mean values for all inputs. The optimistic scenario assumed improved market conditions, including harmonized federal THC testing standards, a 50% increase in processing capacity, a 15% rise in farm-gate prices, and a 10% reduction in per-unit costs. The pessimistic scenario reflected adverse market conditions, including oversupply-driven price drops (–25%), yield declines (–5%), and rising compliance costs (+ 8%). All financial projections were discounted at the prevailing annual discount rate of 5.5% to provide a consistent and defensible evaluation of the expected profitability for floral, grain, and fiber hemp production systems. To evaluate sensitivity to discount-rate risk, the discount rate (r) varied between 4.5% and 6.5%. This provides a robust statistical foundation for assessing the potential economic impacts of hemp production. All statistical analyses were performed using R version 4.3.2 (R Foundation for Statistical Computing). Gaussian copula simulations were carried out using the *copula* package (v1.2–3), distribution fitting was conducted with *fitdistrplus* (v1.1–11), and data visualizations were generated using *ggplot2* (v3.5.1).

## Results and discussion

### Comparative statistics

Table 1 compares hemp production in North Carolina with national averages across several indicators and highlights the shifting structure of hemp production in North Carolina relative to national trends. The results show that North Carolina plays a relatively small role in national floral hemp production but has become a notable contributor to fiber hemp output, reflecting a gradual shift in the region’s production profile after the collapse of the 2019 floral hemp price bubble (Quaicoe et al. 2023). Fiber hemp operations in North Carolina tend to be much larger than the region’s overall farm size, indicating economies of scale and a stronger emphasis on industrial applications.

**Table 1:** Summary statistics of hemp production in North Carolina and the United States

Variable	U.S. Mean	N.C. Mean	N.C. % of U.S
Fiber hemp harvested area (ha)	3,953.92	398.63	10.1
Floral hemp harvested area (ha)	4,671.25	99.15	2.1
Fiber hemp production in the open (kg)	12,319,915.20	2,402,042.80	19.5
Floral hemp production in the open (kg)	6,012,708.80	94,287.80	1.5
Floral production under protection (kg)	94,288.08	4,401.92	4.7
Fiber hemp yield per area (kg·ha⁻^1^)	3,189.75	4,665.07	146.2
Floral hemp yield per area (kg·ha⁻^1^)	1,226.24	1,872.42	72.2
Fiber hemp price (USD·kg⁻^1^)	3.09	5.95	192.9
Floral hemp in the open price (USD·kg⁻^1^)	75.84	93.52	123.3
Floral hemp under protection price (USD·kg⁻^1^)	619.40	931.45	150.4

The estimates are based on data from the USDA National Agricultural Statistical Service

Productivity patterns further differentiate the region from national trends. North Carolina accounts for a relatively small share of national floral hemp production but contributes a more substantial share of fiber hemp output. Table 1 shows that fiber hemp exhibits considerably higher yields per hectare than the national average, suggesting more efficient production practices or favorable agroecological conditions. By contrast, floral hemp yields in the region remain below the national benchmark, reinforcing the relative decline of this sector. Floral hemp has declined in significance, with below-average yields and smaller harvested areas (Table 1), reflecting broader market corrections following the 2019 price bubble (Quaicoe et al. 2023). Farm sizes dedicated to hemp in the region are larger than the average farm size, particularly for fiber hemp. Yield comparisons reveal that fiber hemp performs better in North Carolina than nationally, while floral hemp yields lag (Table 1). The table also highlights price disparities that consistently favor producers. Prices received by growers in the region are substantially higher across both fiber and floral hemp categories, whether grown in open fields or under protection. The higher prices earned by producers across the hemp categories further point to potential comparative advantages in niche markets or value-added supply chains. In summary, North Carolina’s growing emphasis on fiber hemp, combined with its higher yields and price advantages, suggests that North Carolina is emerging as a competitive region for industrial hemp, even if its overall contribution to national production remains modest.

Table 2 contrasts the demographic and occupational characteristics of hemp producers with those of agricultural producers more broadly in the U.S. Although state-level information on operators is unavailable (United States Department of Agriculture 2024), the national comparison provides a benchmark for situating hemp within the wider farm sector, offering insights into whether hemp attracts a distinct subset of producers or mirrors broader trends in U.S. agriculture.

**Table 2:** Characteristics of hemp operators and other agricultural producers in the United States

Variable	U.S. Hemp	U.S. Agriculture
Male (%)	81.3	63.7
Female (%)	18.7	36.3
Average age (years)	49.7	58.1
Farming as a Primary Occupation (%)	49.7	41.9
Other Primary Occupation (%)	50.3	58.1
5 years or less operating any farm (%)	63.0	14.3
6 to 10 years operating any farm (%)	9.7	15.7
11 years or more operating any farm (%)	27.3	70.0
Black or African American Producers (%)	7.0	1.2
White Producers (%)	88.0	95.4
Producers by Other Race (%)	5.0	3.4

The estimates are based on data from the USDA National Agricultural Statistical Service

The results suggest that hemp producers differ in several important ways. They are disproportionately male, notably younger than the average U.S. farmer, with many falling within the 35–54 age range, and relatively inexperienced, with many farming for fewer than five years. These findings correspond with previous studies on hemp enterprises (United States Department of Agriculture 2024; Mooney and Hill 2023; Jenner 2025). This younger demographic is consistent with profile of early adopters in agriculture, who are more likely to embrace new technologies, experiment with emerging crops, and pursue innovative risk management strategies (Diederen et al. 2003; Palm 2020; Läpple and Van Rensburg 2011). At the same time, their relative inexperience may leave them more exposed to volatile prices and underdeveloped markets (Mooney and Hill 2023; Jenner 2025). Another distinctive feature of hemp producers is the higher participation of minority groups compared with the broader U.S. farming population. While White farmers remain the majority, greater representation among Black, Native American, Asian, and multiracial producers (Table 2) suggests that hemp may offer a relatively more accessible entry point into commercial agriculture. These demographic differences, coupled with limited farming experience and the capital-intensive nature of hemp (Jenner 2025), indicate that hemp attracts a distinct set of producers, those more willing to assume risk, innovate, and pursue opportunities outside traditional agricultural pathways. These findings are consistent with previous studies (Mark et al. 2020; Quaicoe et al. 2023; Michels et al. 2025; Dingha et al. 2019), which caution that shifting policies, processing bottlenecks, price volatility, limited marketing channels, and contract uncertainties create substantial risks for hemp farmers.

### Enterprise budget for floral, fiber, and grain hemp production in North Carolina

Table 3 illustrates the stark contrasts in production costs among floral, grain, and fiber hemp enterprises in North Carolina., underscoring how production type shapes both input requirements and financial risk. Floral hemp emerges as the most capital and labor-intensive system, with high expenditures driven by transplanting, skilled labor, and post-harvest processing, as well as additional compliance costs tied to strict regulatory oversight (Table 3). Grain hemp sits at the opposite end of the spectrum, with comparatively modest costs due to minimal input needs, limited processing, and greater reliance on rented land and unskilled labor. Fiber hemp occupies a middle ground, requiring somewhat higher seed and infrastructure investments than grain hemp but remaining far less costly than floral production.

**Table 3:** Estimated per hectare cost of industrial hemp production

	Floral ($. ha⁻^1^)	Grain ($. ha⁻^1^)	Fiber ($. ha⁻^1^)
Transplants	24,709.66	0	0
Seed	0	296.51	691.87
Fertilizer and Lime applied	951.81	267.53	324.68
Soil test	1111.93	24.70	24.709
Plastic mulch	1056.33	0	0
Labor	10,847.54	345.93	345.93
Processing	2421.54	0	0
License Fee	123.54	0	0
Machinery maintenance	1719.19	26.58	26.58
Weed and Pest Control	938.96	69.18	93.89
Other Operational Cost	2611.81	61.77	61.77
Interest on Operating Capital	2372.99	55.62	94.16
Land	12,354.83	247.09	247.09
Building Facilities	17,296.76	86.48	197.67
Equipment and Machinery	12,354.83	49.41	172.96
Irrigation System	3706.44	61.77	148.25
Overhead and Management (15%)	5880.40	147.39	249.54
Total	100,458.63	1574.79	2679.16

The estimates are based on data from USDA National Agricultural Statistical Service

High production costs present a significant challenge for farmers, particularly for smaller operations with limited capital (Michels et al. 2025; Moore et al. 2025). Recent studies emphasize that these substantial start-up expenses can undermine the economic viability of hemp cultivation at a commercial scale (Michels et al. 2025; Patalee et al. 2024). This concern is especially relevant for floral hemp, where intensive labor requirements, specialized inputs, and regulatory compliance contribute to markedly higher costs compared to grain or fiber hemp (Table 3).

These cost patterns highlight that floral hemp, while resource-intensive, aligns with markets offering higher potential returns (Michels et al., 2025; United 2024), making it more suitable for producers with greater access to capital and risk tolerance (Mark et al. 2020; Quaicoe et al. 2023; Michels et al. 2025; Dingha et al. 2019). Conversely, grain and fiber hemp represent lower-cost entry points, but their profitability depends heavily on efficient cost management and access to stable markets (Quaicoe et al. 2023). Importantly, the budgets in Table 3 are illustrative rather than prescriptive, as real-world costs vary with land tenure, local input prices, and labor availability. Moreover, the cost differences observed in North Carolina compared to other hemp-producing states, such as North Dakota (North 2025) and Oregon (Seavert and Crabtree 2021), highlight the importance of regional factors in shaping the competitiveness of hemp enterprises, a finding consistent with previous studies (Budhathoki et al. 2024; Dogbe and Revoredo-Giha 2022; Johnson 2014).

### Expected revenue per hectare across floral, fiber, and grain hemp production types

**Fig. 3:**
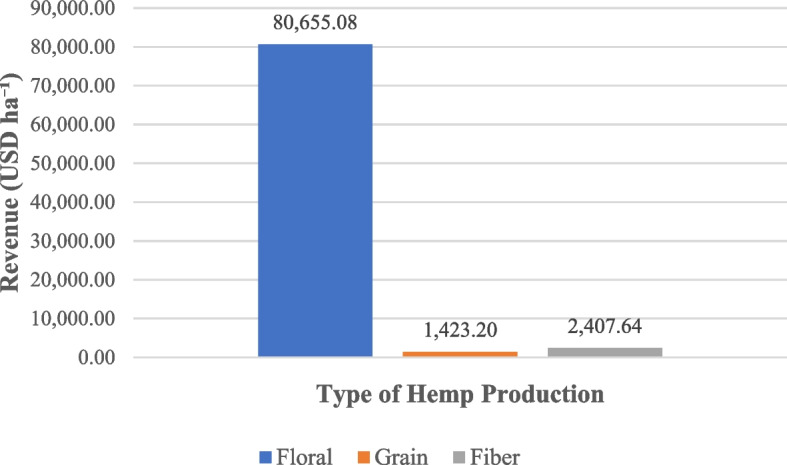
Expected revenue for floral, grain, and fiber hemp. The estimates are based on benchmark price and yield data from USDA National Agricultural Statistical Service

Figure 3 illustrates the relative revenue potential of floral, grain, and fiber hemp enterprises in North Carolina, based on benchmark prices and yields. The results indicate a clear hierarchy of revenue potential, with floral hemp generating the highest expected returns, fiber hemp representing an intermediate position, and grain hemp yielding the lowest returns. Floral hemp’s revenue advantage is largely attributable to its premium market prices and comparatively high yields, as reported in the National Hemp Report (United States Department of Agriculture 2024).

However, these benefits come with elevated risks and high capital requirements, as previously shown in the cost structures of Table 3. Floral markets are highly sensitive to demand fluctuations and to stringent quality and regulatory standards, which magnify financial exposure (Mark et al. 2020; Mark and Will 2019; Antle and Cho 2025; Michels et al. 2025). Grain and fiber hemp, while associated with much lower revenue projections, present leaner cost structures that reduce downside risk and position them for long-term scalability as processing infrastructure and end-use markets expand (Mark et al. 2020; Quaicoe et al. 2023; Patalee et al. 2024). The sharp price collapse of floral biomass in 2019 (Mark et al. 2020), when farmgate values plunged from over 80 USD/kg to below 5 USD/kg (Quaicoe et al. 2023), illustrates the volatility producers must account for when evaluating enterprise viability. Together, these revenue projections highlight the trade-offs between higher-return but risk-intensive floral hemp and lower-revenue but potentially more stable grain and fiber hemp, emphasizing the importance of market diversification (Mark et al. 2020; Michels et al. 2025; Dingha et al. 2019) forward contracting (Mark et al. 2020; Quaicoe et al. 2023), and careful cost management (Antle and Cho 2025; Michels et al. 2025; Moore et al. 2025) in mitigating production risk.

### Net present value, internal rate of return, and benefit‒cost ratio analysis

The NPV analysis involved the use of conservative yield, input price, and product price data to generate estimates for production costs, revenues, and net returns. The NPV was calculated using a 5.5% discount rate, reflecting the prevailing rate by federal reserve at the time of the study. The primary outcomes of the analyses were the simulated distributions of net returns from hemp production for flowers, fiber, and grain. The NPV results indicate that all three types of hemp (floral, grain, and fiber) are likely to generate a positive NPV, although the margins may vary significantly. The positive NPV values in this study suggest that investments in floral, grain, or fiber hemp production are financially viable when evaluated under static assumptions of average yields, production costs, and product prices. In this framework, prices, yields, and costs are held constant at their specified mean levels over the five-year horizon. Consequently, observed differences in long-term outcomes across hemp types reflect the baseline assumptions rather than year-to-year variability or evolving market trends.

Table 4 summarizes the five-year NPV projections for floral, grain, and fiber hemp enterprises. The results indicate a pronounced divergence in long-term economic performance across hemp types. Floral hemp emerges as the enterprise with a consistently positive and substantial NPV, reflecting the combined effect of high market prices and revenue streams that outweigh its elevated production costs. Grain hemp shows modest but sustained profitability, suggesting that while margins are relatively thin, the enterprise can remain economically viable under stable market and production conditions. Fiber hemp, by contrast, exhibits only marginal returns over the projection period, underscoring the financial challenges of competing in a market with low prices and limited value-added opportunities.

**Table 4:** Five-year net present value projection (USD ha⁻^1^)

Year	Floral	Grain	Fiber
0	–100,459	–1,575	–2,679
1	30,130	419	663
2	29,130	405	641
3	27,888	388	614
4	26,170	364	576
5	24,929	346	549
Total	37,788	369	365

The estimates are based on benchmark price and yield data from USDA National Agricultural Statistical Service

The estimated IRRs for floral, grain, and fiber hemp were 18%, 13%, and 10%, respectively. As these IRRs exceed the federal discount rate of 5.5%, they further confirm that hemp cultivation, regardless of the intended product, flowers, grains, or fibers, could represent a sound investment opportunity. The estimated BCR’s for floral, grain, and fiber hemp production were 1.5, 1.2, and 1.1, respectively, indicating that each dollar spent on production generates more than a dollar in revenue. This underscores the economic viability and relatively low risk of cultivating hemp for flowers, grain, or fiber. While positive NPVs, IRRs, and BCRs suggest the likelihood of a favorable outlook for investment in hemp production, it is important to note that the long-term profitability of these ventures is heavily influenced by the stability and success of the hemp market across the value chain. Collectively, these results reinforce the central role of crop type in shaping the financial sustainability of hemp cultivation and highlight the need for careful alignment of production strategies with market realities.

These findings are broadly consistent with prior studies on the feasibility of hemp production, which highlight substantial regional variability in profitability and emphasize the strong influence of regional conditions and production systems on hemp’s economic viability (Antle and Cho 2025; Michels et al. 2025; Barnes et al. 2023; Moore et al. 2025; Patalee et al. 2024; Dogbe and Revoredo-Giha 2022; Johnson 2014). For instance, Michels et al. (2025), highlighted that German farmers perceive profitability primarily when hemp cultivation is integrated into diversified operations with reduced input costs—a model more consistent with grain and fiber hemp in North Carolina than with capital-intensive floral hemp. Similarly, Barnes et al. (2023) demonstrated that intercropping fiber hemp with loblolly pine in Southwestern U.S. can yield returns 25% higher than monocropping. This illustrates the potential of strategic integration and diversification as key to enhancing farm-level profitability and lower-value hemp uses. In Oregon, Antle and Cho (2025) reported wide variability in outcomes. Their findings suggest that even at relatively low prices, hemp could be profitable for 10%–25% of farms, particularly where supply chains and production systems were favorable. These findings parallel North Carolina’s case for grain and fiber hemp, where modest NPVs could become more attractive under favorable market development and policy support.

Conversely, studies such as Patalee et al. (2024) argue that small-scale hemp operations are unlikely to be viable because of high start-up costs—an observation particularly relevant for floral hemp in North Carolina, where profitability depends on substantial capital resources and risk tolerance. Moore et al. (2025) likewise identified production costs as a critical threat to farmer profitability across U.S. hemp systems. Overall, while North Carolina’s positive NPVs confirm hemp’s potential as a commercially viable crop, the contrast between floral, grain, and fiber hemp align with broader global and regional evidence that hemp can be financially viable under favorable structural and market conditions. However, they also highlight that profitability is highly context-dependent and hinges on structural factors such as input costs, scale, diversification strategies, and market development.

### Sensitivity analysis

To assess the robustness of profitability under different external conditions, a sensitivity analysis was conducted using three deterministic scenarios—baseline, optimistic, and pessimistic—over a five-year horizon with a 5.5% discount rate. The baseline scenario reflects average observed conditions for prices, costs, and yields, while the optimistic and pessimistic scenarios introduce adjustments to capture potential regulatory, market, and weather shocks. The optimistic scenario, which incorporates harmonized THC testing standards, a 50% expansion in processing capacity, a 15% increase in farm gate prices, and a 10% reduction in unit production costs, substantially enhances profitability. Relative to the baseline, NPVs per hectare increase by 38.6% for floral hemp, 282.6% for grain hemp, and 314.5% for fiber hemp. By contrast, the pessimistic scenario integrates an 8% rise in compliance costs, a 25% decline in farm gate prices, and a 5% reduction in yields due to weather variability. These adverse conditions reduce NPVs by 118.3%, 73%, and 45.3% for floral, grain, and fiber hemp, respectively.

**Fig. 4:**
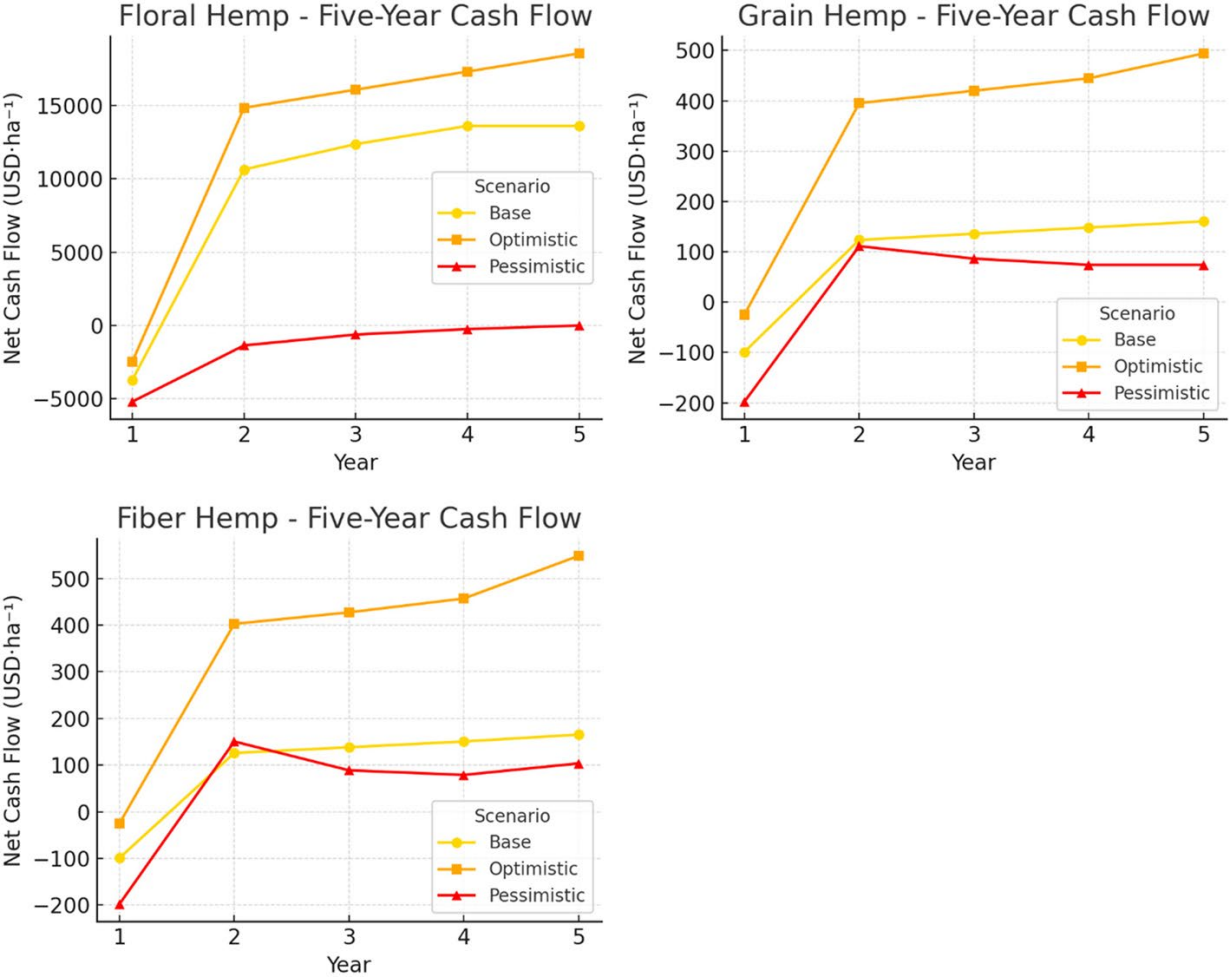
Five-year per hectare cash flow projection for floral, grain, and fiber hemp. The estimates are based on benchmark price and yield data from USDA National Agricultural Statistical Service

Figure 4 illustrates the annual net cash flow trajectories across scenarios. The results underscore the high sensitivity of floral hemp to market and regulatory conditions.

While it can generate substantial profits under favorable assumptions, it also exhibits significant downside risk, particularly in the early years when initial investments drive negative cash flows. Grain hemp, although less profitable overall, displays greater resilience, with narrower differences between optimistic and pessimistic outcomes. Fiber hemp occupies an intermediate position, offering moderate profitability with relatively limited volatility compared to floral hemp, making it a more balanced though less lucrative option. Additional sensitivity testing with respect to the discount rate (varied between 4.5 and 6.5%) indicates that NPVs for floral hemp fluctuate by less than ± 10%, and profitability rankings across hemp types remain unchanged. This suggests that the conclusions regarding relative financial performance are robust to reasonable variations in the cost of capital.

When contextualized within the broader literature, these results are consistent with Antle and Cho’s (2025) ex-ante evaluation of hemp in Oregon, which emphasizes that profitability hinges on hemp prices, production costs, and supply chain capacity, with only 10–25% of farms achieving profitability under lower price conditions. Their findings further stress that adoption into existing production systems will remain limited if the industry remains small under ongoing regulatory and economic uncertainty, which constrains market size and depresses farm gate prices. According to Antle and Cho’s (2025), higher price levels than those observed after the “bust” years of 2020–2021 are required to make hemp economically competitive with established crops. The importance of market infrastructure in determining profitability is echoed across multiple studies. Cherney and Small (Cherney and Small 2016) argue that hemp cannot be profitable without viable processing capacity, while Barker (2020) emphasizes that the industry’s long-term viability depends on infrastructure development. Mark et al. (2020) similarly identify processing bottlenecks, shifting policies, and price volatility as key barriers to entry. Sterns (2019) highlights the risks of limited marketing channels and contract uncertainty, findings reinforced by surveys of U.S. farmers (Dingha et al. 2019; Moore et al. 2025; Stevenson 2017). Beyond the U.S., Michels et al. (2025) report from Germany that profitability is strongly linked to diversification strategies and input reduction, whereas Patalee et al. (2024) highlight the prohibitive role of high start-up and compliance costs, particularly for small-scale operators. Compared with these regions, the North Carolina case reveals a sharper divergence between optimistic and pessimistic outcomes, especially for floral hemp. This suggests both a heightened exposure to volatility and a comparatively greater upside potential under favorable market and regulatory conditions.

### Monte Carlo simulation outcomes

The Monte Carlo simulation builds on the deterministic sensitivity analysis by quantifying the range of possible profitability outcomes under stochastic variability in yields, prices, and costs. Consistent with the scenario analysis, floral hemp emerges as the most profitable system on average, but also the most volatile (Table 5). The wide dispersion of simulated outcomes underscores its dual character: high upside potential under favorable market and regulatory conditions, but substantial downside risk when adverse shocks occur. By contrast, grain and fiber hemp exhibit considerably lower expected profitability (Table 5), yet their narrower outcome ranges indicate relatively more stability and reduced exposure to extreme losses.

**Table 5:** Monte Carlo simulation of profitability for floral, grain, fiber hemp (USD ha⁻^1^)

	Floral	Grain	Fiber
Mean	31,886	432	717
Standard. deviation	10,506	178	284
5th percentile	–11,119	–148	–272
95th percentile	70,661	1,013	1,694
Coefficient of Variation	0.33	0.41	0.40
Mean Difference: Floral vs Grain	31,454***		
Mean Difference: Floral vs Fiber	31,169***		
Mean Difference: Fiber vs Grain	285***		

The estimates are based on benchmark price and yield data from USDA National Agricultural Statistical Service

*** = statistical significance at 1% (*p* < 0.01)

The coefficients of variation confirm moderate to high instability across all hemp systems, with floral hemp’s profitability swings being most pronounced. Pairwise tests of mean profitability further demonstrate that floral hemp significantly outperforms both grain and fiber hemp, while fiber offers a modest but statistically significant advantage over grain. These results echo the deterministic analysis, which highlighted the strong contrast between optimistic and pessimistic outcomes in North Carolina, particularly for floral hemp. Overall, the deterministic and stochastic analyses suggest that while floral hemp offers superior expected returns, its viability hinges on producers’ capacity to manage higher financial risk and withstand market volatility (Antle and Cho 2025; Michels et al. 2025; Barnes et al. 2023; Moore et al. 2025; Patalee et al. 2024; Dogbe and Revoredo-Giha 2022; Johnson 2014). Grain and fiber hemp, although less lucrative, present more stable opportunities and may be more accessible to producers seeking lower-risk entry points. The comparative stability of these systems suggests that diversification across hemp types, or integration into broader crop portfolios, could help mitigate risk exposure at the farm level (Michels et al. 2025).

## Conclusion

This study evaluated the economic feasibility of cultivating floral, grain, and fiber hemp in North Carolina using enterprise budgets, discounted cash flow analysis, and risk-based modeling approaches. The results reveal several key insights. Production costs vary considerably, with floral hemp requiring the highest per-hectare investment due to intensive inputs, labor, and regulatory compliance, while grain and fiber hemp maintain lower cost structures. Investment appraisal metrics (NPV, IRR, BCR) suggest that all three systems are financially viable under baseline conditions, though floral hemp, while the most profitable, demands greater capital and entails higher financial exposure. Sensitivity analysis and stochastic simulations show that profitability is highly responsive to price and yield variability. Floral hemp offers the greatest return potential but also the highest downside risk, whereas grain and fiber provide more modest yet comparatively stable outcomes. At the industry level, North Carolina’s underdeveloped pricing mechanisms and limited processing capacity heighten producer vulnerability to market volatility. Overall, this study provides the first comprehensive enterprise budgets and risk-adjusted profitability estimates for hemp in North Carolina. Floral hemp delivers the highest returns but with substantial risk, fiber offers moderate profitability with balanced risk, and grain generates the lowest but most stable returns. These results underscore the trade-offs facing producers and the need for improved processing infrastructure, market transparency, and supportive policy to ensure long-term sector sustainability.

### Limitations of the study

While this study provides valuable insights into the economic viability of industrial hemp production in North Carolina, few limitations should be noted. First, the analysis was constrained by limited data availability. Consequently, the simulation models relied on a narrow set of historical data, which may reduce the precision of profitability estimates. Future research using more comprehensive datasets could improve the robustness of the findings and provide stronger guidance for policy and industry development. Second, the economic models applied in this study were based on static assumptions of prices, yields, and costs across a five-year period. While appropriate given the short production history of hemp, this approach does not capture year-to-year variability or dynamic market adjustments. The results should therefore be interpreted as indicative benchmarks rather than predictive forecasts. Third, the analysis is specific to North Carolina and reflects its unique production costs, regulatory context, and market conditions. Because land, labor, input, and processing costs differ across regions, the generalizability of the results is limited. Finally, while the sensitivity analysis incorporated weather-related yield shocks, regulatory compliance costs, and farm-gate price volatility, it did not address all potential sources of uncertainty, such as changes in labor availability, varietal yield differences, or unanticipated supply chain disruptions. Future research should consider these additional risk factors to better assess the long-term resilience of hemp production systems.

## Data Availability

Data for the study were sourced from the U.S. Department of Agriculture (USDA)-NASS.

## Abbreviations

BCR: Benefit–cost ratio
CBD: Cannabidiol
IRS: Increasing returns to scale.
IRR: Internal rate of return
ha: Hectare
kg: Kilogram
NPV: Net present value
N.C: North Carolina
R: R statistical software
THC: Tetrahydrocannabinol
USD: United States dollar
U.S.: United States
USDA: United States Department of Agriculture
CAGR: Compound Annual Growth Rate

## Declarations

The authors declare that the manuscript is original.
